# Altered Homotopic Connectivity in the Cerebellum Predicts Stereopsis Dysfunction in Patients With Comitant Exotropia

**DOI:** 10.3389/fnhum.2022.917769

**Published:** 2022-06-02

**Authors:** Fei Chen, Zhirou Hu, Hui Liu, Fangyuan Zhen, Chenlu Liu, Qiuming Li

**Affiliations:** ^1^Department of Ophthalmology, The First Affiliated Hospital of Zhengzhou University, Zhengzhou, China; ^2^Department of Ophthalmology, Jiangxi Provincial People’s Hospital, Nanchang, China

**Keywords:** comitant exotropia, functional magnetic resonance imaging, voxel-mirrored homotopic connectivity, functional connectivity, support vector machine

## Abstract

**Purpose:**

Comitant exotropia (CE) is a common eye disorder characterized by impaired stereoscopic vision and eye deviation. Previous neuroimaging studies demonstrated that patients with CE were accompanied by specific functional and structural abnormalities of the brain. However, the effect of impaired stereoscopic vision and eye deviation on interhemispheric homotopic connectivity remains unknown.

**Methods:**

A total of thirty-six patients with CE (25 males and 11 females) and 36 well-matched healthy controls underwent magnetic resonance imaging scanning. The voxel-mirrored homotopic connectivity (VMHC) method was applied to assess the interhemispheric homotopic connectivity changes in patients with CE. Furthermore, the support vector machine method was applied to assess to differentiate patients with CE from healthy controls (HCs) with the VMHC maps as a feature.

**Results:**

Compared with HCs, patients with CE showed significantly increased VMHC values in the bilateral cerebelum_ 8 and cerebelum_4_5. Moreover, we found that the VMHC maps showed an accuracy of 81.94% and an area under the curve of 0.87 for distinguishing the patients with CE from HCs.

**Conclusion:**

Our study demonstrates that patients with CE showed interhemispheric homotopic connectivity changes in the cerebellum, which might reflect the neurological mechanisms of impaired stereoscopic vision and eye deviation in patients with CE.

## Introduction

Comitant exotropia (CE) is a common eye disease characterized by ocular deviation and stereopsis dysfunction. The prevalence of exotropia is approximately 1% in all children (Govindan et al., 2005). There are several risk factors for the development of strabismus, including myopia (Zhang et al., 2021), anisometropia (Smith et al., 2017), and a family history of strabismus (Taira et al., 2003). Stereopsis dysfunction is a core clinical symptom in patients. Binocular visual function in patients with strabismus can interfere with daily life and work. Moreover, patients with strabismus may also have mental health problems (Lin et al., 2014; Lee et al., 2022). Strabismus surgery is currently an important tool in the treatment of strabismus. However, some patients are unable to re-establish stereopsis after strabismus surgery. The integration of binocular visual information occurs in the lateral geniculate nucleus; this integration leads to binocular vision and depth perception. However, the neural mechanisms that underlie central nervous system dysfunction in patients with CE remain unclear.

Functional magnetic resonance imaging (fMRI) has been widely used to study changes in brain neural activity in patients with strabismus. Chen et al. (2021a) reported that brain neural activity considerably differed between patients with CE and HCs; affected areas included the middle occipital gyrus and supplementary motor area/precentral gyrus, implying that stereopsis dysfunction was present in patients with CE. Tan et al. (2018) reported that patients with CE exhibited abnormal network activity in various brain regions. Using a voxel-based analysis method, Yan et al. (2010) showed that patients with CE had lower fractional anisotropy values in the right middle occipital gyrus and right supramarginal gyrus. Huang et al. (2018) reported that patients with CE had increased resting cerebral blood flow in the supplementary eye field, cingulate eye field, and frontal eye field. Jin et al. (2022) also found that, compared to healthy controls, patients with CE had abnormalities in the cerebellar, sensorimotor, auditory, and visual networks. Thus, the abovementioned studies demonstrated that patients with CE had abnormalities in vision and eye-motor control regions of the brain (supplementary eye field, cingulate eye field, and frontal eye field). However, the effects of impaired stereopsis and eye deviation on interhemispheric homotopic connectivity remain unclear. The human brain exhibits interhemispheric homotopic connectivity. Homotopic connectivity within the visual cortex participates in visual information processing (Dougherty et al., 2005; Butt et al., 2015). Furthermore, homotopic connectivity may reflect the mechanisms that underlie central nervous system disorders. The voxel-mirrored homotopic connectivity (VMHC) method, first proposed by Zuo et al. (2010), quantifies the homotopic connectivity between individual voxels in one hemisphere and their mirror voxel counterparts in the other hemisphere. Previous studies demonstrated that the VMHC method has a good spatial and temporal resolution (Yang et al., 2019; Chen et al., 2021b; Wu et al., 2021). Although there have been many studies concerning the functional connectivity changes that occur in CE, the effects of impaired stereopsis and eye deviation on interhemispheric homotopic connectivity in patients with CE are still unknown. Furthermore, with the rapid development of machine learning technology, MRI-related machine learning methods are increasingly used in disease classification; such applications can provide sensitive biomarkers for disease diagnosis and prediction. Support vector machine (SVM) methods are supervised machine learning algorithms widely used for MRI data classification; these methods can achieve individual-level classification and detect biomarkers based on neuroimaging data. SVM methods can reduce the high dimensionality of MRI data and improve the level of data generalization. Compared with other machine learning methods, such as random forest, naïve Bayes, and convolutional neural network. SVM methods have been successfully used to classify diseases, such as iridocyclitis (Tong et al., 2021), primary dysmenorrhea (Gui et al., 2021), and major depressive disorder (Gao et al., 2021). However, no studies have used SVM and VMHC methods to distinguish patients with CE from HCs.

Considering the published findings described above, the purpose of this study was to determine whether patients with CE exhibit interhemispheric homotopic connectivity. Additionally, an SVM method was used to investigate the predictive value of VMHC data for diagnostic classification. Thus, we hypothesized that the patients with CE might be associated with abnormal interhemispheric homotopic connectivity.

## Materials and Methods

### Participants

Thirty-six patients with CE (mean age, 24.25 ± 1 years) and 36 healthy controls (HCs; mean age, 24.46 ± 1.31 years) were recruited from the first hospital affiliated with Zhengzhou University and Jiangxi Provincial People’s Hospital. The diagnostic criteria of patients with CE were: (1) CE, exodeviation angles between 15Δ and 80Δ; (2) without a history of strabismus surgery; The exclusion criteria of CE individuals in the study were: (1) additional ocular-related complications (e.g., cataract, glaucoma, high myopia, or optic neuritis); (2) sensory exotropia, fixed exotropia, secondary exotropia, restricted exotropia, and exotropia with vertical exotropia; and (3) concomitant exotropia were associated with amblyopia.

### Ethics Statement

All research methods followed the Declaration of Helsinki and were approved by the Ethical Committee for Medicine of the first hospital affiliated with Zhengzhou University and Jiangxi Provincial People’s Hospital. Participants enrolled in the study of their own accord and were informed of the purpose, methods, as well as potential risks before signing an informed consent form.

### Magnetic Resonance Imaging Acquisition

Magnetic resonance imaging scanning was performed on a 3-tesla magnetic resonance scanner (Discovery MR 750W system; GE Healthcare, Milwaukee, WI, United States), with the eight-channel head coil. Functional images were obtained by using a gradient-echo-planar imaging sequence. All the subjects were asked to keep their eyes closed and relaxed without falling asleep.

### Functional Magnetic Resonance Imaging Data Analysis

All preprocessing was performed using the toolbox for Data Processing and Analysis of Brain Imaging (DPABI)^1^ (Yan et al., 2016) which is based on Statistical Parametric Mapping (SPM12)^2^ implemented in MATLAB 2013a (MathWorks, Natick, MA, United States) and, briefly, using the following steps: (1) Remove first ten volumes; (2) Slice timing effects, motion-corrected; (3) Normalized data (in Montreal Neurological Institute 152 space) to EPI template were re-sliced at a resolution of 3 mm × 3 mm × 3 mm; (4) Spatial smoothing by convolution with an isotropic Gaussian kernel of 6 mm × 6 mm × 6 mm full width at half maximum; (5) The fMRI data with linear trends were removed; (6) Linear regression analysis was applied to regress out several covariates (mean framewise displacement, global brain signal, and averaged signal from cerebrospinal fluid and white matter); and (7) Temporal band-pass was filtered (0.01–0.08 Hz) to reduce the influence of noise.

### Voxel-Mirrored Homotopic Connectivity Analysis

To evaluate the interhemispheric connectivity, the VMHC method was performed using the DPABI toolkit according to previous research (Zuo et al., 2010). The VMHC values for each participant were calculated as the Pearson correlation between each pair of symmetrical interhemispheric voxels’ time series.

### Support Vector Machine Analysis

To evaluate whether the VMHC metrics alterations could serve as potential diagnostic metrics for CE, we performed ML analyses using the SVM algorithm with the average VMHC values of all clusters showing significant intragroup differences as the features. The following steps were followed (Schrouff et al., 2013): (1) The VMHC maps of two groups served as a classification feature; and (2) Then, the leave-one-out cross-validation technique was applied to perform SVM classifier validation. This procedure was repeated n times. For classification, two classes were defined (patients’ group and HCs group) and processed using a soft-margin hyper-parameter approach. The soft-margin parameters take the values 0.01, 0.1, 1, 10, 100, and 1,000 in the SVM classifier in the current version of PRoNTo, which make the model obtain the maximum interval hyperplane with the minimum classification error. To quantify the performance of classification methods, accuracy, sensitivity, and specificity were reported. Besides the classification accuracy, the receiver operating characteristic (ROC) curves and the corresponding area under the curve (AUC) were also computed to evaluate the classification efficiency.

### Statistical Analysis

The independent sample *t*-test was used to investigate the clinical features of the two groups.

The one-sample *t*-test was conducted to assess the group mean of VMCH maps. The two-sample *t*-test was used to compare the two group differences in the VMCH maps using the Gaussian random field (GRF) method (two-tailed, voxel-level *P* < 0.01, GRF correction, cluster-level *P* < 0.05).

## Results

### Demographics and Disease Characteristics

There were no statistically significant differences between the CE and HC groups in gender or age, the results of these clinical data were summarized in Table 1.

**TABLE 1:** Demographics and visual measurements between two groups.

Condition	CE group	HC group	*T*-values	*P*-values
Gender (male/female)	(25/11)	(25/11)	N/A	N/A
Age (years)	15.80 ± 2.46	16.00 ± 2.68	–0.240	0.812
Handedness	36 R	36 R	N/A	N/A

*Note: Independent t-test for the other normally distributed continuous data (means ± SD). Abbreviations: CE, comitant exotropia and HC, health control.*

### Different Voxel-Mirrored Homotopic Connectivit Values Between Two Groups

**FIGURE 1:**
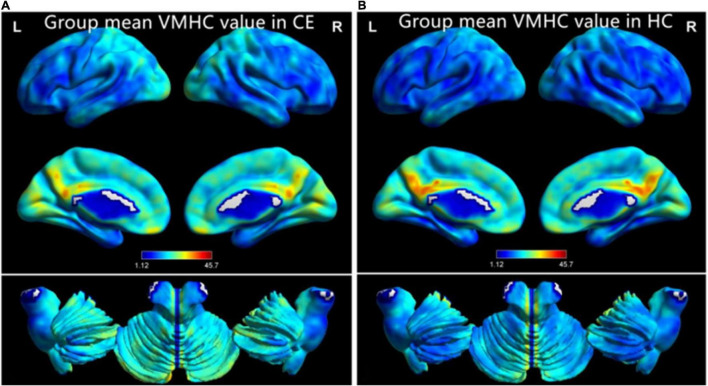
One-sample *t*-test results of VMHC maps within comitant extropia (CE) group **(A)** and HC group **(B)**. Abbreviations: CE, comitant exotropia; HC, health control; and VMHC, voxel-mirrored homotopic connectivity.

**FIGURE 2:**
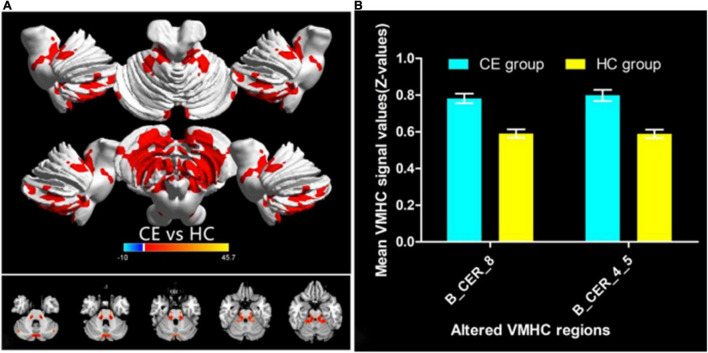
Significant VMHC differences in the CE and HC group **(A)**; the mean of altered VMHC values between patients with CE and HCs **(B)**. Abbreviations: CE, comitant exotropia; HC, health control; VMHC, voxel-mirrored homotopic connectivity; and CER, cerebellum.

The group means of VMHC maps of the CE and HC are shown (Figures 1A,B). Compared with the HCs, patients with CE showed significantly increased VMHC values in the bilateral cerebelum_8 and bilateral cerebelum_4_5 (Table 2 and Figure 2A). The mean values of altered VMHC values were shown with a histogram (Figure 2B).

**TABLE 2:** Different voxel-mirrored homotopic connectivity (VMHC) values between two groups.

Condition	Brain regions	BA	Peak *T*-scores	MNI coordinates (*x*, *y*, *z*)	Cluster size (voxels)
CE>HC	Bilateral cerebelum_ 8	–	4.8927	±6 –63 –51	697
CE>HC	Bilateral cerebelum_4_5	–	4.8512	±12 –33 –30	154

*Note: Different VMHC values between two groups (voxel-level P < 0.01, GRF correction, cluster-level P < 0.05). Abbreviations: CE, comitant exotropia; HC, health control; MNI, Montreal Neurological Institute; and VMHC, voxel-mirrored homotopic connectivity.*

### Support Vector Machine Classification Results

**FIGURE 3:**
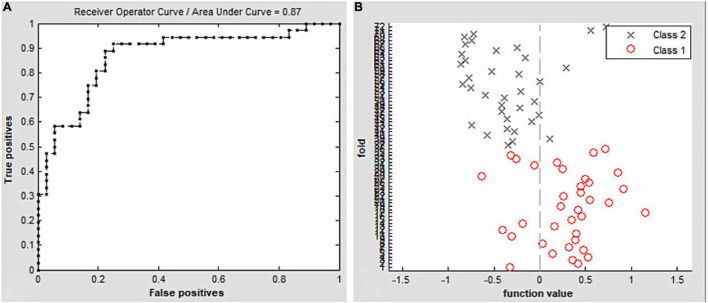
Classification results using support vector machine (SVM) based on VMHC values. Function values of two groups (class 1: CE group; class 2: HC group); **(A)**. The ROC curve of the SVM classifier with an AUC value of 0.87 **(B)**. Abbreviations: CE, comitant exotropia; HC, health control; VMHC, voxel-mirrored homotopic connectivity; ROC, receiver operating characteristic; SVM, support vector machine; and AUC, area under the curve.

To evaluate the classification ability of the SVM model, the accuracy, sensitivity, specificity, and precision were calculated and the ROC curve of the classifier was illustrated in Figure 3. Moreover, we found that the VMHC maps showed an accuracy of 81.94% and an AUC of 0.87 for distinguishing the patients with CE from HCs.

## Discussion

Compared to HCs, patients with CE had significantly greater VMHC values in the bilateral cerebellum_8 and cerebellum_4_5. Additionally, the VMHC maps exhibited an accuracy of 81.94% and an AUC of 0.87 for distinguishing patients with CE from HCs.

In this study, patients with CE exhibited a significant increase in VMHC values in the cerebellum. The cerebellum plays an important role in balance control and motor function; it is also involved in eye movement and visual perception. Thier and Markanday (2019) demonstrated that the cerebellum plays an important role in visually guided eye movements and visual motion perception. Moreover, the cerebellum is responsible for encoding eye movements (Lixenberg et al., 2020). Thus, the vermal cortex to visual motion perception is non-motor and involves cerebellar influence on cortical information processing. Previous neuroimaging studies have shown that strabismus is accompanied by cerebellar dysfunction. Zhu et al. (2018) demonstrated a significant reduction in functional connectivity between the left Brodmann area (BA17) and the left lingual gyrus/posterior cerebellar lobe in patients with CE. Ouyang et al. (2017) found that the left posterior cerebellar gray matter volume (GMV) was decreased in patients with CE. Higher VMHC reflects the increased functional connectivity in both hemispheres indicating the hyperconnectivity of bilateral brain areas. Besides, the higher VMHC value in the cerebellar might reflect the high functional connectivity within the cerebellar network. Consistent with these findings, we found a significant increase in cerebellar VMHC values in patients with CE, which suggests that oculomotor and visual perception dysfunction are present in patients with CE.

Our study showed that using VMHC maps, the SVM method could distinguish patients with CE from HCs with an accuracy of 81.94% and an AUC of 0.87. The SVM method has been widely used in machine learning studies of MRI imaging. To our knowledge, no study has evaluated the combined effect of VMHC and SVM techniques on CE classification. In our study, we investigated whether VMHC maps could be used as a classification feature to distinguish these groups, based on a machine learning approach that used an SVM classifier. We found that good machine classification accuracy could be achieved using VMHC maps. Thus, our results suggest that VMHC maps may be suitable for distinguishing patients with CE from HCs.

However, some limitations should be acknowledged. First, our sample size was small. Second, the clinical course of CE varies among patients; this may have affected the accuracy of our results. Third, the VMHC maps were based on BOLD signals, which may be affected by physiological noise. Finally, we applied the SVM method to assess the sensitivity of the classification; this method might have been affected by the sample size.

## Conclusion

Our findings indicate that cerebellar homotopic connectivity is increased in patients with CE, thereby providing insight into the neural mechanism that underlies oculomotor and visual perception dysfunction in patients with CE. Furthermore, VMHC maps may be suitable for distinguishing patients with CE from HCs.

## Data Availability Statement

The original contributions presented in the study are included in the article/supplementary material, further inquiries can be directed to the corresponding authors.

## Ethics Statement

The studies involving human participants were reviewed and approved by The First Affiliated Hospital of Zhengzhou University, Jiangxi Provincial People’s Hospital. The patients/participants provided their written informed consent to participate in this study.

## Author Contributions

FC, ZH, and HL contributed to data collection, statistical analyses, and writing the manuscript. FZ and CL designed the protocol and contributed to the MRI analysis. FC and QL designed the study, oversaw all clinical aspects of study conduct, and manuscript preparation. All authors contributed to the article and approved the submitted version.

## Conflict of Interest

The authors declare that the research was conducted in the absence of any commercial or financial relationships that could be construed as a potential conflict of interest.

## Publisher’s Note

All claims expressed in this article are solely those of the authors and do not necessarily represent those of their affiliated organizations, or those of the publisher, the editors and the reviewers. Any product that may be evaluated in this article, or claim that may be made by its manufacturer, is not guaranteed or endorsed by the publisher.

## Footnotes

1
http://www.rfmri.org/dpabi

2
http://www.fil.ion.ucl.ac.uk
